# Application of image-recognition techniques to automated micronucleus detection in the in vitro micronucleus assay

**DOI:** 10.1186/s41021-024-00305-9

**Published:** 2024-04-24

**Authors:** Hiromi Yoda, Kazuya Abe, Hideya Takeo, Takeji Takamura-Enya, Ayumi Koike-Takeshita

**Affiliations:** 1 Biomedical Research Center, Kanagawa Institute of Technology, 1030 Shimo-Ogino, Atsugi, Kanagawa 243-0292 Japan; 2 Department of Applied Biosciences, Kanagawa Institute of Technology, Atsugi, Japan; 3 Department of Electrical and Electronic Engineering, Kanagawa Institute of Technology, Atsugi, Japan; 4 Department of Applied Chemistry, Kanagawa Institute of Technology, 1030 Shimo-Ogino, Atsugi, Kanagawa 243-0292 Japan

## Abstract

**Background:**

An in vitro micronucleus assay is a standard genotoxicity test. Although the technique and interpretation of the results are simple, manual counting of the total and micronucleus-containing cells in a microscopic field is tedious. To address this issue, several systems have been developed for quick and efficient micronucleus counting, including flow cytometry and automated detection based on specialized software and detection systems that analyze images.

**Results:**

Here, we present a simple and effective method for automated micronucleus counting using image recognition technology. Our process involves separating the RGB channels in a color micrograph of cells stained with acridine orange. The cell nuclei and micronuclei were detected by scaling the G image, whereas the cytoplasm was recognized from a composite image of the R and G images. Finally, we identified cells with overlapping cytoplasm and micronuclei as micronucleated cells, and the application displayed the number of micronucleated cells and the total number of cells. Our method yielded results that were comparable to manually measured values.

**Conclusions:**

Our micronucleus detection (MN/cell detection software) system can accurately detect the total number of cells and micronucleus-forming cells in microscopic images with the same level of precision as achieved through manual counting. The accuracy of micronucleus numbers depends on the cell staining conditions; however, the software has options by which users can easily manually optimize parameters such as threshold, denoise, and binary to achieve the best results. The optimization process is easy to handle and requires less effort, making it an efficient way to obtain accurate results.

**Supplementary Information:**

The online version contains supplementary material available at 10.1186/s41021-024-00305-9.

## Introduction

The in vitro micronucleus test is a widely used genotoxicity test and is the first choice for detecting chromosomal aberrations induced by clastogenic, heterologous, and aneugenic chemicals [1–5]. The test can be performed within a short assay time of approximately 2 days, and the results are relatively easy to interpret [3]. The test method is available worldwide based on the guidelines provided by the organization for economic cooperation and development [3]. A micronucleus (MN) is a whole or fragment of a chromosome that is not incorporated into the main nucleus during cell division. This occurs when genotoxic substances interact with chromosomes and cause chromosomal disruption. In vitro, tests that combine micronucleus and bacterial mutation assays performed using mammalian cell lines have been found to be highly correlated with the results of in vivo studies on carcinogenicity and genotoxicity [1, 6].

The micronucleus test results are calculated based on the number of cells that form micronuclei (MN cells). For this, the number of MN cells present in 1,000 intact interphase cells per dish is counted under a microscope. However, this task becomes more time-consuming as the number of samples increases. Several improved methods, such as image analysis, laser scanning cytometry, and flow cytometry, have been proposed to avoid errors in the scoring process owing to human observation and to increase the speed of cell counting [7–11]. Among these, MN detection by flow cytometry is a rapid analytical method, and studies have explored ways to improve MN selectivity and detection thresholds, including the optimization of staining dyes [9, 12]. Several researchers have also reported computer-assisted image analysis of MN counts using commercial and/or open-source software [13–16].

CellProfiler (Broad Institute) is a free cell image analysis software that can be used for applications like MN detection and detection of binucleated cells formed in cytokinesis-block micronucleus (CBMN) assays [9]. This automated system was developed to reduce analysis time and cost. However, this free software has a wide variety of features that allow various analyses of cell images. So, it is necessary to find the best image processing method for the micronucleus test from wealthy functions, set the pixel size for cytoplasm, cell nuclei, micronuclei, and cell debris, one by one, and perform these detections in sequence without failure. ImageJ is a free software that can analyze images [17]. However, it has fewer functions than CellProfiler, and users must begin by searching for macros for various uses.

Therefore, in this study, we developed an easy-to-operate micronucleus/cell detection application specialized for micronucleus testing.

This application was developed on the Windows 10 operating system, and we ensured that the application could run on an even previous-generation PC. Therefore, this software provides easy, one-click counting of cell counts and MN cell counts from micronucleus test images, regardless of PC generation.

## Materials and methods

### Materials

Mitomycin C (1 mg/mL), methyl methanesulfonate, hydrogen peroxide (30%), and potassium chromate were obtained from Nacalai Tesque (Kyoto, Japan). Unless otherwise stated, all commercially available reagents were used without further purification.

### Methods

#### Cells

CHL/IU cells were obtained from the Japanese Collection of Research Bioresources and were maintained in Dulbecco’s Modified Eagle Medium (DMEM) supplemented with 10% fetal bovine serum inactivated by pre-heating and with 1% of Penicillin (10,000 units/mL)-streptomycin (10,000 µg/mL). For the in vitro micronucleus test, an uncoated glass slide that had been washed with ethanol and dried was placed at the bottom of a new φ10-cm polystyrene dish, and 11 mL of culture medium was added to cover the glass slide. CHL/IU cells were seeded at a concentration of 1 × 10^5^ cells. The cells were incubated at 37 °C in a 5% CO_2_ atmosphere for 3 days before adding the test chemicals.

#### Sample treatment and slide preparation

After seeding 10^5^ CHL/IU cells and culturing them for 3 days, the medium was replaced with a medium containing the following dose chemicals: hydrogen peroxide (H_2_O_2_) at concentrations of 3.75, 7.5, 15, and 30 µg/mL [18]; potassium chromate (K_2_CrO_4_) at concentrations of 1.2, 2.4, and 4.9 µg/mL [19]; mitomycin C (MMC) at concentrations of 0.0125, 0.025, 0.05, 0.1, and 0.2 µg/mL [18]; and methyl methanesulfonate (MMS) at concentrations of 10, 20, 40, and 80 µg/mL [18]. After 24 h of exposure to the medium containing chemicals, the medium was replaced with fresh medium without chemicals and the cells were cultured for an additional 24 h. As MMC, MMS, and H_2_O_2_ are micronucleus-positive without metabolically active substances such as liver homogenate supernatant S9 [18], micronucleus tests were performed without S9. Control cells, which served as blank, were treated in the same manner without any additives.

The glass slide was carefully removed from the dish, rinsed twice with PBS using a staining tray (Kartell, part number 1-1413-02), and immersed in 2% paraformaldehyde in PBS for 15 min at room temperature to fix the cells. The cells were then rinsed with PBS and permeabilized by immersion in a 0.1% Triton X-100/PBS solution for 5 min at room temperature. A slide glass was removed from the staining tray and placed on a flat surface, and an acridine orange (AO) solution (160 µg/mL in PBS) was added dropwise to spread over the surface of the fixed cells. The excess staining solution was removed by covering the slide with a cover glass.

#### Microscopic observation and photography

Glass slide samples dyed with acridine orange were observed under a CKX41 inverted microscope (Olympus; 20x objective lens) equipped with LED fluorescence modules LED-FL-BG/MI and URFLT50 and a light source. The cells were observed under blue light and photographed using a microscopic digital camera DP80 equipped with the cellSens software (Olympus) set with a sensitivity of ISO 400 and an exposure time of 40 ms. Each slide was photographed over 50 times at various microscope fields of view, and each image was serially numbered and saved in the RGB-tiff format.

#### Automated image analysis by the micronucleus (MN) /cell detection software

Automatic analysis of captured color images of cells begins by dropping a folder containing multiple images onto the user interface of the micronucleus detection software. The RGB images were first separated into three colors: red, green, and blue, and the blue images were excluded because they were not used for analysis. Nuclei containing micronuclei were observed as green spots, and the cytoplasm appeared red. All separated colors were transformed into grayscale. To detect cell nuclei and micronuclei, a reduced-size image of the entire image in green was generated. When the images before and after the size reduction of the green image were superimposed, the points where the green color disappeared after reduction were detected as micronuclei, and the points where the brightness appeared as a double circle after reduction were detected as cell nuclei.

To determine the location and area of the cytoplasm, a green image of the cell nucleus and micronuclei and a red image of the cytoplasm were superimposed to create a new image in which the entire cytoplasm (including the nuclei) was filled with one color.

**Fig. 1:**
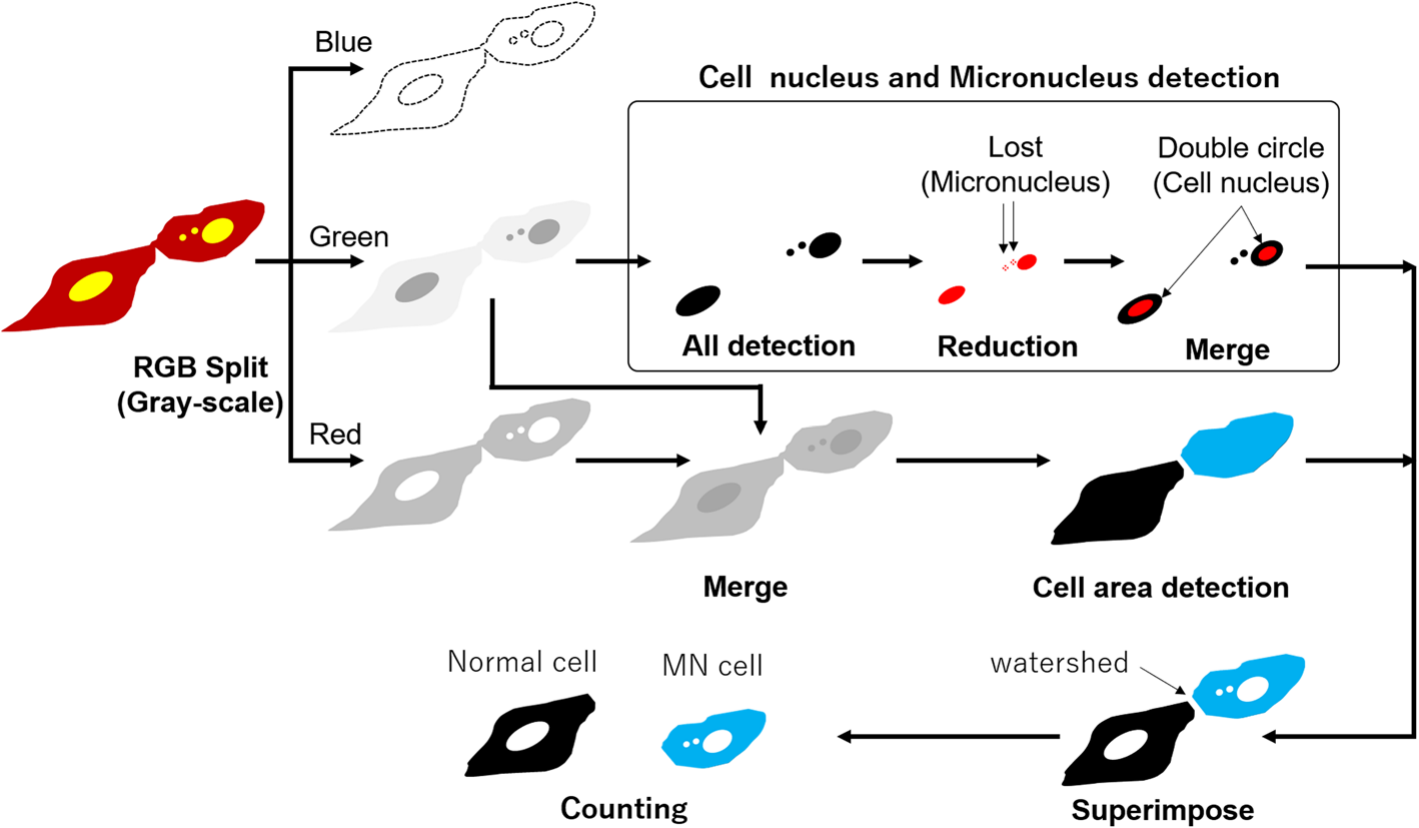
Scheme of processing to detect normal cells and MN cells from microscopic images

Following this, green images of the cell nuclei were superimposed onto the generated images of the cytoplasm. To separate adjacent cells, cell boundaries located near the midpoint between adjoining cell nuclei were cut using watershed processing. After determining the position of the cytoplasm, the nuclei and micronuclei were superimposed on top of the cytoplasm. If only one cell nucleus was present in one cytoplasm, it was considered a normal cell, and if there were one or more micronuclei in addition to the cell nucleus, it was considered an MN cell. This process is illustrated in Fig. 1.

To automatically detect the cytoplasm, cell nuclei, and micronuclei, this software uses six thresholds: binarization threshold (binarization TH), gradation center value, large and small kernel size, noise removal, and micronucleus processing threshold (micronucleus TH). The binarization TH is a parameter that roughly detects cell nuclei by extracting and binarizing only the green image from the RGB image. For automatic analysis, the maximum continuous value was determined from the histogram of the green image of the test sample images, and five specified values (65, 75, 85, 120, and 165) were set as the representative binarization THs. These values are based on the relationship between the maximum value of the histograms of the images used in the development and the practical threshold value. The maximum value and the threshold value are directly related, i.e., a higher maximum value results in a higher threshold value and vice versa. The gradation center value specifies the intermediate value of the gradation conversion process, which combines red and green images and emphasizes the cytoplasm using a sigmoid function. Values above this center value are highlighted, and those below are suppressed. Processing that emphasizes the cytoplasmic region is effective for edge detection of the cytoplasm, which tends to be blurred. The size of the kernel indicates the filter size for enlarging/reducing cell nuclei and micronuclei and was used to fill in empty holes and remove minute parts during binarization. Because the kernel size is used to detect micronuclei through a process of contraction and expansion, the detection performance depends on the kernel size. The noise removal threshold prevents the detection of a size smaller than the micronucleus that remains when a microscopic region is extracted. Areas smaller than this value are considered as noise and removed. Micronucleus TH represents a detection threshold based on the circularity of micronuclei. When the circularity is one, only perfectly round micronuclei are detected. However, the value depends on the resolution because the images are represented as pixels. In the image analysis using the software, the initial analysis obtained parameter values, followed by reanalysis, which was performed by adjusting the parameter values for each image (individual reanalysis) or target images all at once with arbitrary parameter values (batch reanalysis).

The MN/cell detection software consists of a program folder containing the Main.exe file written in Python and Bio_Count.exe, which serves as the user interface, and files written in Microsoft Visual Basic Scripting Edition (VBScript). Because this software uses VBScript, it can be executed on Windows OS (Windows 10 and 11). In this study, image analysis was performed using a laptop computer with a Core i7 10510U CPU, 8GB RAM, and Windows 10 Home 64bit OS.

#### Manual analysis of images

MN cells were visually counted using the same images used for automated image analysis. This study did not classify cells by the shape of their nuclei, as in the CBMN method [8], because it determined the number of micronuclei in cells.

#### *T*-test

To apply the t-test, more than 1000 cells were measured per sample by manual counting and automated image analysis of the microscopic images. The counted cells were the total cells in each dose of the chemical, and the number of NM cells was counted. The micrographs used for the image analysis and manual counting were identical. Student’s *t*-test (one-sided test) was used as an independent two-sample test, assuming the use of the micronucleus detection software in the actual micronucleus test.

## Results

### Acquisition of counting values by image analysis

**Fig. 2:**
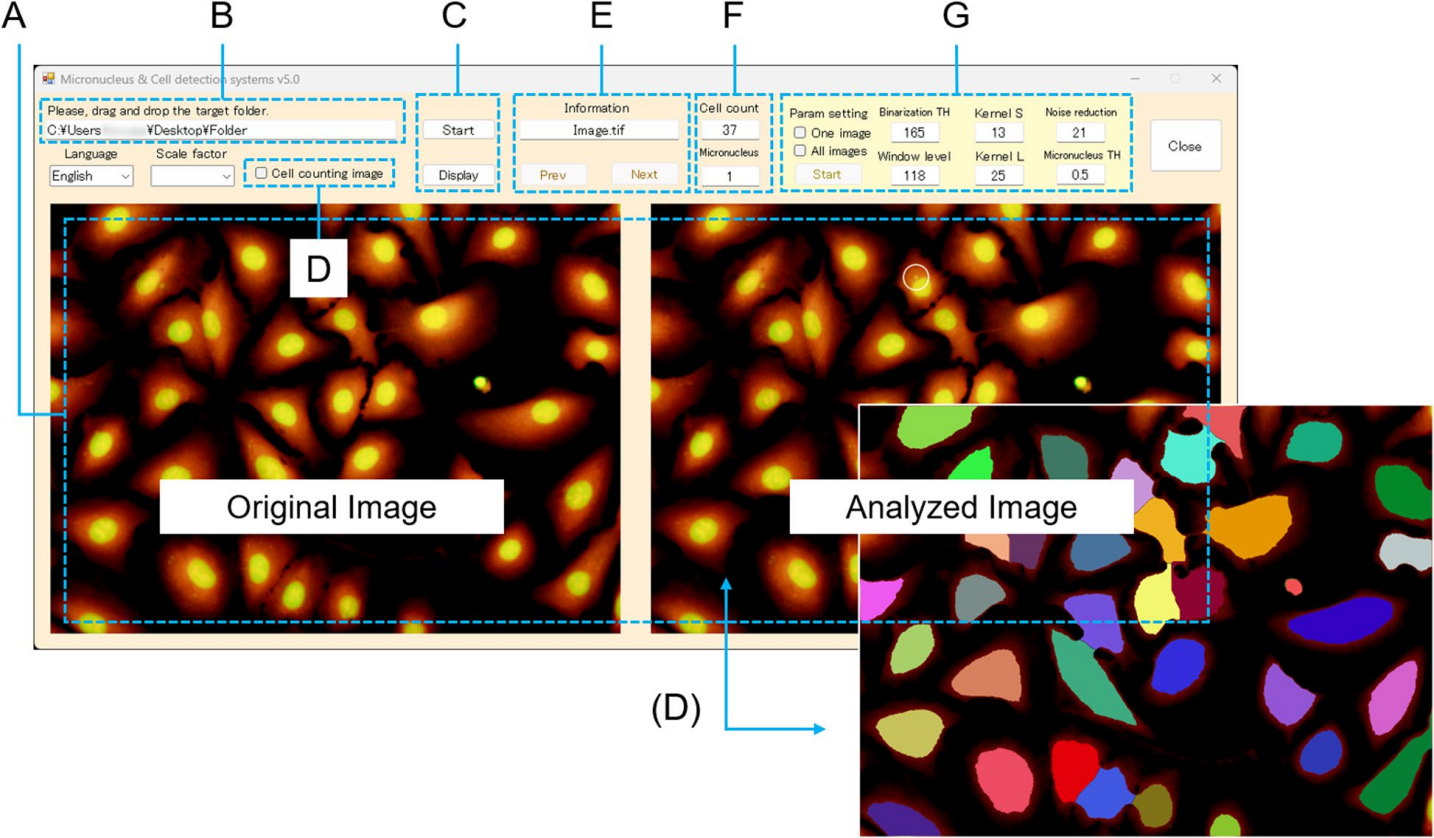
Micronucleus detection application interface. **A **Display area for pre-analysis image (left) and post-analysis image, (**B**) image folder name, (**C**) button to start image analysis and display analyzed image, (**D**) check button to switch images of detected cytoplasm and micronuclei, (**E**) button to select analyzed image, (**F**) small window to display number of analyzed cells and micronuclei-containing cells, and (**G**) individual mode area for adjusting micronucleus detection parameters. In this figure, the position of detected cells on the analyzed image is shown

**Fig. 3:**
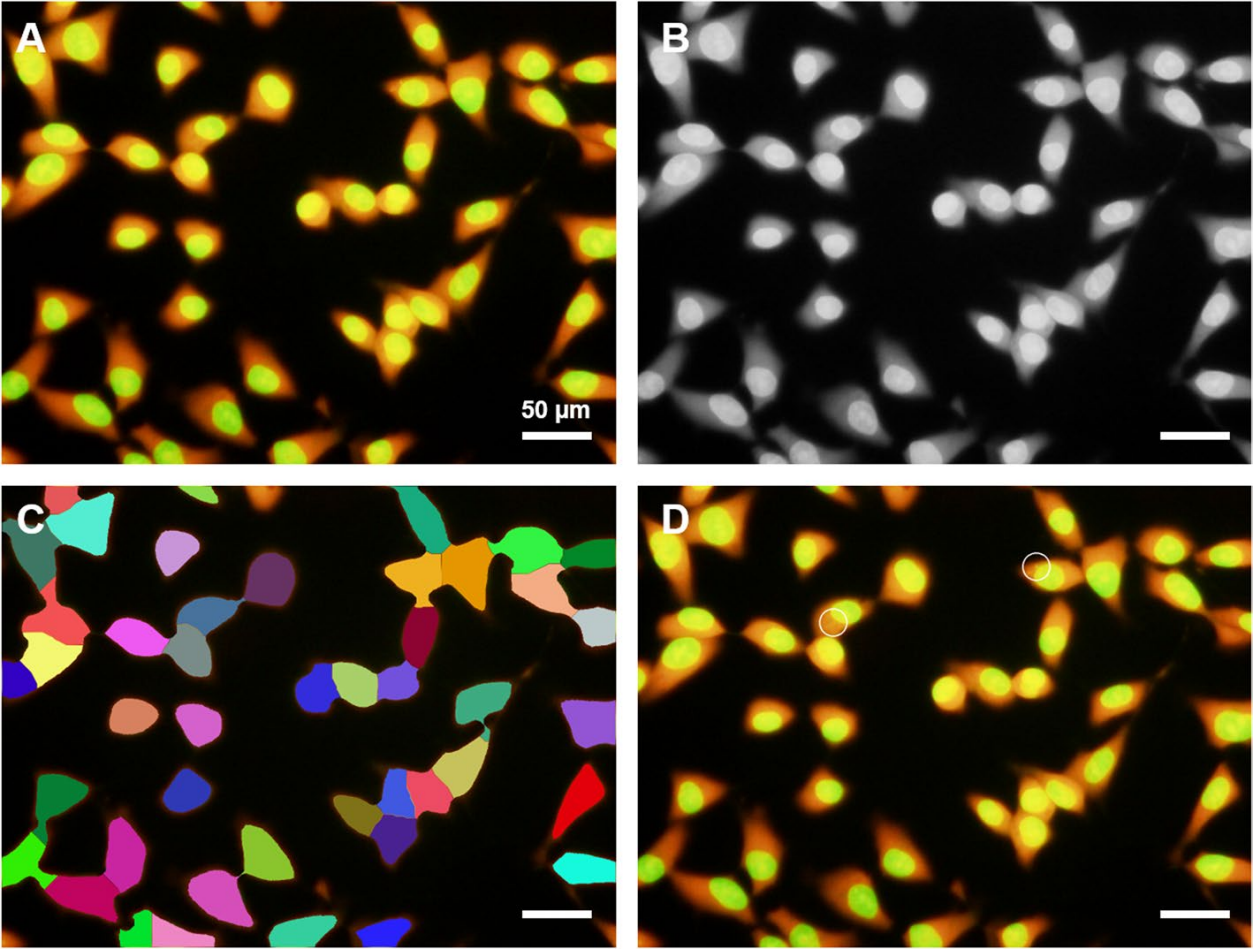
Images generated by the image analysis application. **A** Original image, (**B**) grayscale, (**C**) total cells, and (**D**) MN cells of the K_2_CrO_4_-treated cells. All micrograph bars showed 50 µm

The micronucleus/cell detection software can be placed in any folder on the Windows OS. However, the folder containing the microscope images to be analyzed should be placed on the Windows OS desktop or one level below it, such as My documents, My pictures, or other such folders. After executing the Bio_Count. exe run in the software program folder to open the micronucleus/cell detection software, the image folder was dragged and dropped into the software (Fig. 2A). The folder and image to be analyzed appear as a small window on the upper left side (Fig. 2B). The software starts to explore when the start button is clicked (the display button becomes inactive simultaneously), and the display button becomes active when the analysis has ended (Fig. 2C). After the analysis starts, six folders are created in the software program folder: Image, Grayscale, Count, Shokaku, Text, and Palam. The Image folder stores the original image for display (Fig. 3A). The Grayscale folder holds a green image constructed from the original RGB image (Fig. 3B). Although the grayscale image is not displayed in the software, it is saved as an intermediate image during the analysis of the cell nuclei and micronuclei to be checked at any time. The Count folder contains an image showing the detected cytoplasm in different colors (Fig. 3C). The Shokaku folder stores color images of detected and undetected micronuclei generated during the analysis. If a micronucleus is detected, the image shows a mark surrounded by a white line at the detected position (Fig. 3D). The Text folder stores a text file in which the numbers of detected cells and micronuclei are shown. The Palam folder stores text files containing the parameters for each analyzed image. There are six parameters: the binarization TH, the kernel size (S and L) to adjust the size of cell nuclei and MNs to be detected, the threshold of noise size to be removed (noise reduction), the MN detection threshold based on the roundness (micronucleus TH), and the window level to emphasize the brightness and darkness of the image, and a text file containing these values created for each image.

When the display button is clicked after analysis, the left panel displays the original image stored in the Image folder, and the right panel displays the image stored in the Shokaku folder or the cell mark image stored in the Count folder (Fig. 2). Additionally, the file name of the image being displayed (Fig. 2E) and the number of cells and cells with MN (MN cells) automatically detected from the image (Fig. 2F) were displayed simultaneously. Figure 2G shows the parameter values assigned for the analysis of this image. Individual reanalysis or batch reanalysis after the initial analysis could be performed by changing the parameter values displayed in this area. In the individual reanalysis, the parameter values for each image acquired during the initial analysis were changed on the software’s user interface in the image mode. Subsequently, reanalysis was performed on a single image. Batch reanalysis was performed by overwriting the parameter values in the following order: binarization TH, kernel S, kernel L, noise reduction, micronucleus TH, and window level in the PalamLock.txt file, which was automatically created in the Palam folder during the initial analysis. Overwritten parameter values can be used when the image modes are at the user interface.

### Results of manual counting of chemical-induced MN cells

Table 1 shows the number of cells and MN cells obtained by manual counting. The incidence of micronuclei in control cells was 0.71%. Cells treated with H_2_O_2_ showed a micronucleus incidence of 2.61% at 3.75 µg/mL, 5.26% at 7.5 µg/mL, 7.52% at 15 µg/mL, and 9.56% at 30 µg/mL. Cells treated with K_2_CrO_4_ showed a micronucleus incidence of 2.66% at 1.2 µg/mL, 6.34% at 2.4 µg/mL, and 10.94% at 4.9 µg/mL. Micronuclei generation of 3.23% at 0.0125 µg/mL, 4.15% at 0.025 µg/mL, 13.58% at 0.05 µg/mL, 39.24% at 0.1 µg/mL, and 19.36% at 0.2 µg/mL in cells treated with MMC rate. In cells treated with MMS, the micronucleus incidence was 4.58% at 10 µg/mL, 9.24% at 20 µg/mL, 18.56% at 40 µg/mL, and 4.33% at 80 µg/mL.

**Table 1:** Comparison of the count results of the manual counting and the MN/cell detection software

Sample	(µg/mL)	Number of photographs	Manual counting			Cell/MN counting software									Cell viability
						Initial analysis			Individual reanalysis			Batch reanalysis			
			Total cells	MN cells	MN	Total cells	MN cells	MN	Total cells	MN cells	MN	Total cells	MN cells	MN	
			(n)	(n)	(%)	(n)	(n)	(%)	(n)	(n)	(%)	(n)	(n)	(%)	(%)
Control	0	45	1555	11	0.71	1548	13	0.84	1549	16	1.03	1593	23	1.44	100
H_2_O_2_	3.75	55	1340	35	2.61	1352	155***	11.46	1352	32	2.37	1352	25	1.85	71
	7.5	57	1292	68	5.26	1336	91*	6.81	1297	72	5.55	1334	60	4.50	66
	15	43	1250	95	7.60	1262	93	7.37	1262	93	7.37	1262	93	7.37	84
	30	79	2040	189	9.26	2053	257*	12.52	2055	197	9.59	2093	197	9.41	75
K_2_CrO_4_	1.2	50	1353	36	2.66	1354	30	2.22	1354	31	2.29	1348	32	2.37	78
	2.4	60	1436	91	6.34	1408	87	6.18	1408	89	6.32	1413	84	5.94	69
	4.9	66	1270	139	10.94	1280	98*	7.66	1274	129	10.13	1299	118	9.08	56
MMC	0.0125	49	1919	62	3.23	1901	76	4.00	1896	68	3.59	1910	78	4.08	113
	0.025	36	1661	69	4.15	1679	112**	6.67	1676	72	4.30	1688	62	3.67	134
	0.05	30	1465	199	13.58	1456	225	15.45	1456	198	13.60	1497	183	12.22	141
	0.1	80	1203	472	39.24	1172	464	39.59	1172	455	38.82	1182	448	37.90	44
	0.2	65	1684	326	19.36	1682	378	22.47	1688	319	18.90	1704	321	18.84	75
MMS	10	49	1658	76	4.58	1642	80	4.87	1639	68	4.15	1647	90	5.46	98
	20	50	1147	106	9.24	1145	115	10.04	1143	116	10.15	1123	113	10.06	66
	40	73	1347	250	18.56	1363	257	18.86	1359	239	17.59	1361	257	18.88	53
	80	187	1155	50	4.33	1136	135***	11.88	1136	52	4.58	1149	58	5.05	18

MN (%) meant the percentage of micronucleus-containing cells in the total cells

^*^: *p* < 0.05, **: *p* < 0.01, ***: *p* < 0.005 showed significant differences between the number of detections by the MN/cell detection software and the number of detections by manual counting in the same chemical concentration image group by Student’s t-test

Cell samples treated with H_2_O_2_ (30 µg/mL), MMC (0.1 µg/mL), and MMS (40 and 80 µg/mL) required 70 or more images for analysis because the number of cells present in the field of view was low. In such cases, the incidence of micronuclei was high. The relative survival percentage was lowest at 80 µg/mL of MMS (18%), followed by 10 µg/mL of MMC (44%), 40 µg/mL of MMS (44%), and 4.9 µg/mL of K_2_CrO_4_ (56%), indicating the tendency of survival to decrease with a higher concentration of the administered chemicals (Table 1, Fig S1).

### Total cell numbers counting by image analysis

The binarization TH, which was the parameter showing the greatest variation among the samples, depended on the color of the cells stained with acridine orange (Table S1). The binarization THs for the sample images acquired during the initial analysis were 65, 75, 85, 120, or 165. It was noted that the more apparent the color difference between the cytoplasm and cell nucleus, the higher the binarization of TH (Fig S2). For the other parameters, the kernel size was automatically determined based on the size of the cell nuclei and micronuclei contained in the photographed cells, and the noise removal value was automatically selected according to the kernel size. Fixed values of 0.5 and 118 were used as parameters for the micronucleus TH and grayscale intermediate value, respectively.

**Fig. 4:**
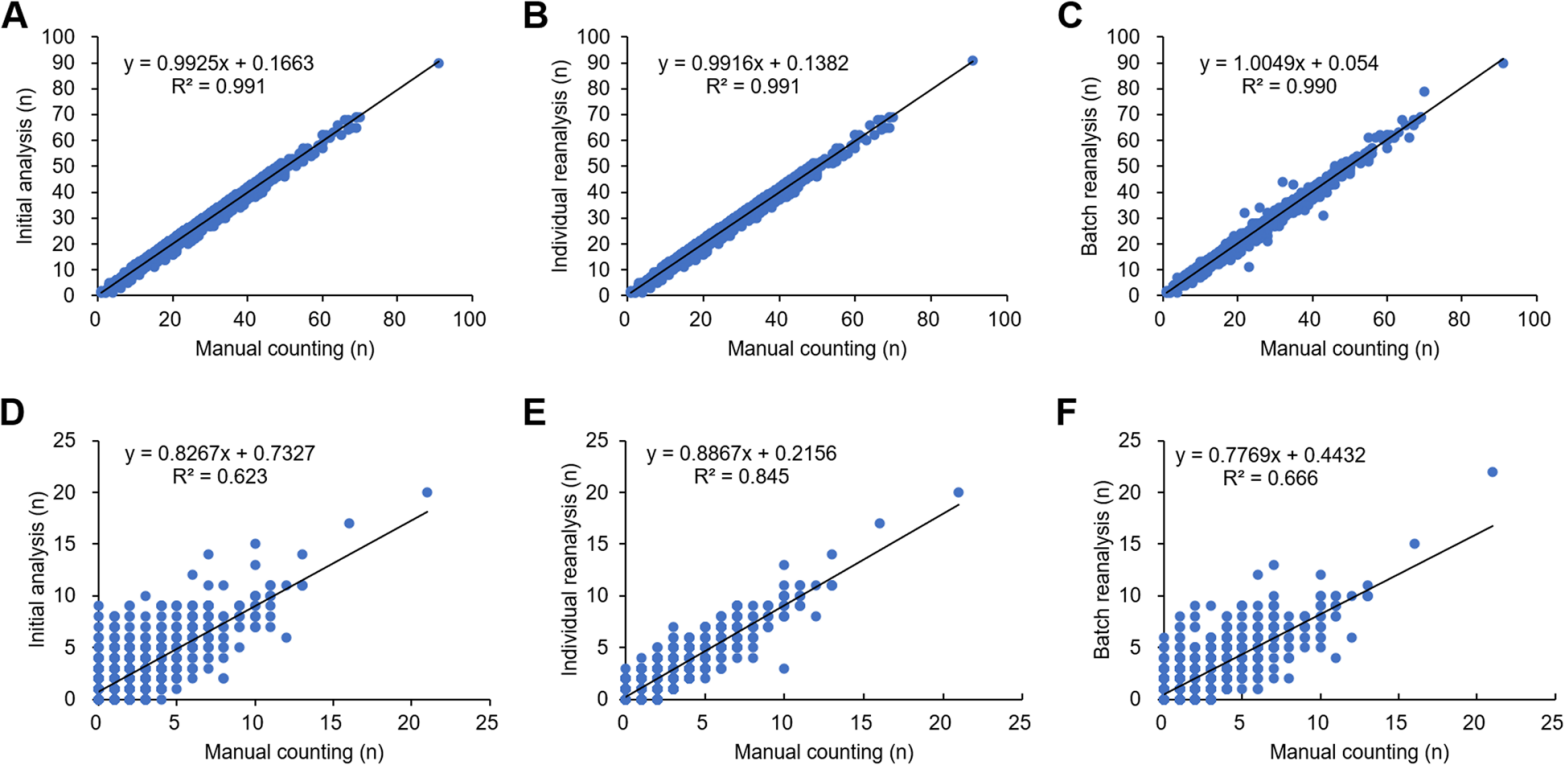
The correlation graph of the total cells and NM-cells counting by the manual and the cell/MN counting software. (**A**, **B**, and **C**) Total cell counts and (**D**, **E**, and **F**) NM cell counts are done using the cell/MN counting software and manual counting. **A**, **D** The initial analysis, (**B**, **E**) the manual parameter adjustment of the re-analysis, and (**C**, **F**) the batch processing of the re-analysis by the MN/cell detection software

Figure 4 presents a set of correlation charts showing the level of matching between the counts obtained manually and using the MN/cell detection software for the same image. Manual counting and initial analysis using the counting software for the total cells in 1074 images showed a good correlation (Fig. 4A), indicating only minor differences between manual counting and image analysis by the software. After the initial analysis, reanalysis was performed by manually adjusting the parameter values for one image at a time on the software screen (individual reanalysis), while reanalysis of all images at the same time was performed by fixing six parameter values (batch reanalysis). In both reanalyses using the counting software, the total cell counts for each tested chemical dose were consistent with those obtained with manual counting (Fig. 4B and C), and the difference between the two methods was within 50 cells for all samples.

### MN detection by image analysis

The MN/cell detection software counted the detected micronuclei as MN cells if their locations overlapped the cytoplasmic regions. In the initial analysis, the NM cell counts of each the tested chemical dose were close to the manual counting results for control, 15 and 30 µg/mL of H_2_O_2_, 1.2 and 2.4 µg/mL of K_2_CrO_4_, 0.0125, 0.05, 0.1, and 0.2 µg/mL of MMC and 10, 20, and 40 µg/mL of MMS in the images. On the other hand, compared with manual counting, approximately 1.3- to 2.7-fold more MN cells were counted in images of cell samples treated with 3.75 µg/mL and 7.5 µg/mL H_2_O_2_, 4.9 µg/mL K_2_CrO_4_, 0.025 µg/mL MMC, and 80 µg/mL MMS. Student’s *t*-test (one-sided test) showed significant differences in the results between manual counting and the MN/cell detection software in these images, with significant differences in MN cell numbers (Table S2). The correlation coefficient between the number of MN cells counted in the initial analysis, which contained false-positive NMs, and the number of NM cells by manual counting was approximately 0.6 (Fig. 4D).

Following the initial analysis, an individual reanalysis was performed in which the parameter values of each image were manually adjusted. The threshold values for cell image binarization TH and MN detection (Micronucleus TH) depended on the staining state of the cells and the resolution of the photographic image; therefore, visual confirmation was necessary for the correct selection of the MN.

**Fig. 5:**
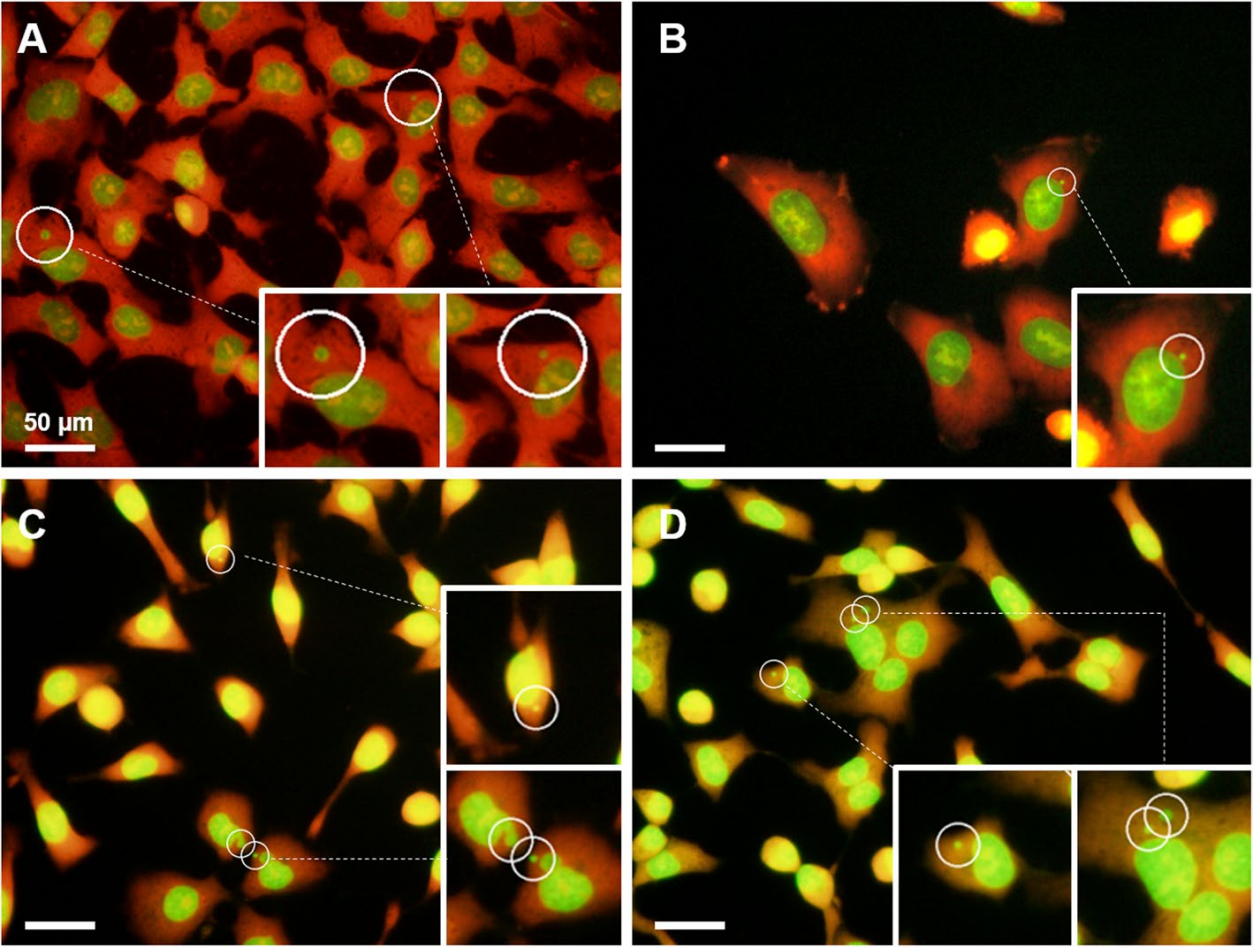
Image in which MN cells are correctly detected by the optimal binarization threshold using the MN/cell detection software. Examples of the image binarization values are (**A**) 85, (**B**) 120, (**C**) 165, and (**D**) 170

Examples of images in which MN cells were detected at the optimal binarization TH are shown in Fig. 5. The cells in Fig. 5A were stained excessively red by AO, and yellow granules were visible inside the green-stained cell nuclei. As the micronuclei in the figure are the same green as the cell nuclei, MN cells were detected by lowering the binarization TH to 85. As shown in Fig. 5B and C, micronuclei were very close to the cell nuclei; however, MN cells were detected at binarization THs of 120 and 165, respectively. The cells in Fig. 5D had poor AO staining and overall high green color intensity; however, MN cells were detected by increasing the binarization TH to 170.

**Fig. 6:**
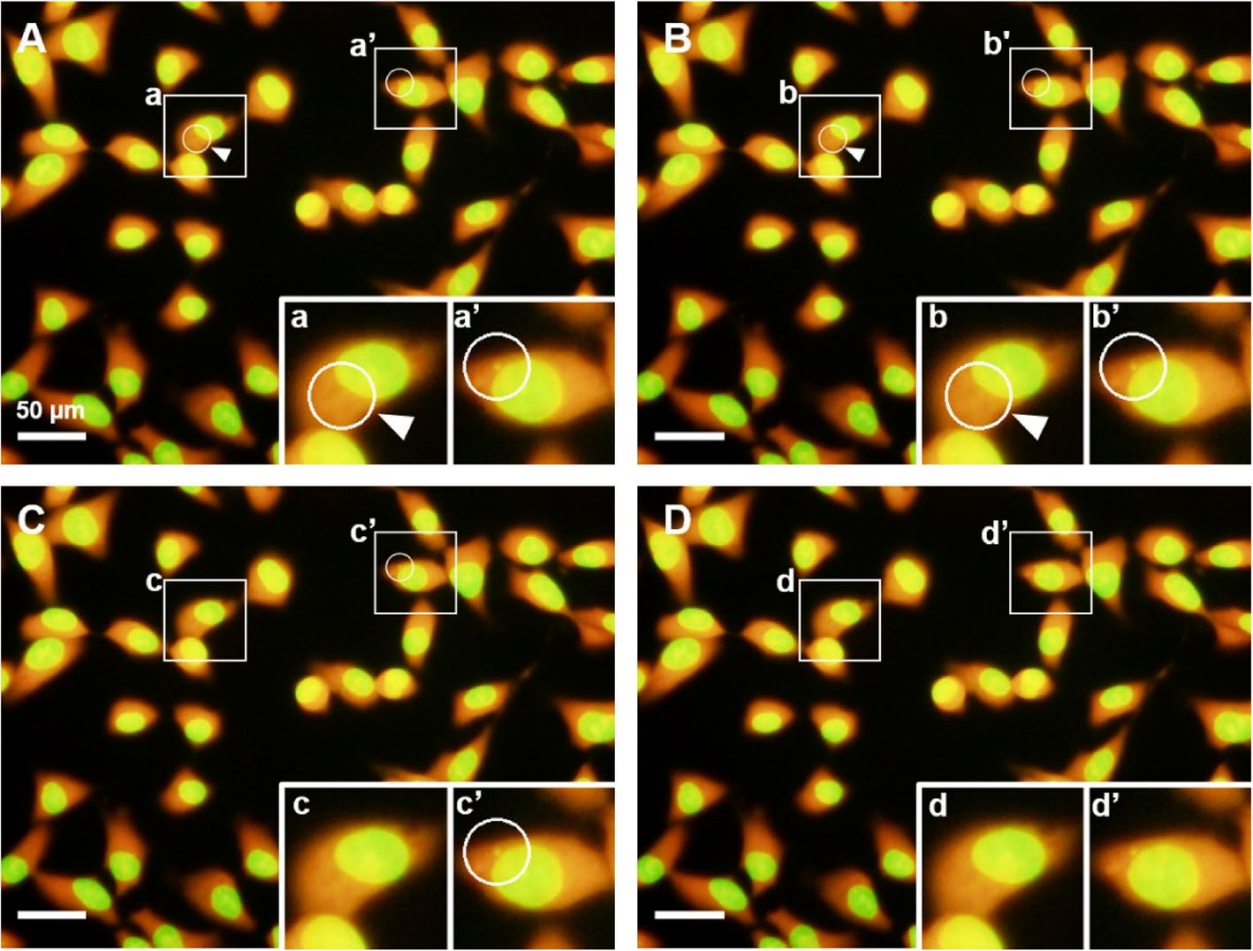
Micronucleus detection results corrected in individual mode. The threshold for micronucleus detection is at the values of (**A**) 0.5, (**B**) 0.6, (**C**) 0.65, and (**D**) 0.7. Small letters and prime letters indicated the enlarged view of each position. (**A**, **B**) The inappropriate threshold. White arrows pointed to a white-lined circle where the MN was not presented (White arrows were displayed by the author). (**C**) The optimum value of the threshold. (**D**) Inappropriate threshold. Even the detected MN was lost

 A similar reanalysis was performed for the software detection of MN cells. In the MN/cell detection software, the MN detection threshold is essential for detecting the MN. Figure 6 shows an example of the individual reanalysis of the images of K_2_CrO_4_-treated cells with the MN detection threshold changed in the range of 0.5 to 0.7. When the MN detection threshold was 0.5 and 0.6, the software detected excess micronuclei even in the cytoplasm of cells that had no micronuclei (Fig. 6A and B). However, at 0.65, one micronucleus was correctly detected (Fig. 6C); at 0.7 (Fig. 6D), micronuclei were no longer detected. Individual reanalysis was performed on images with misplaced MN cells, as shown in the initial analysis. The values of Micronucleus TH were generally in the range of 0.6–0.7, and the Binatization TH generally ± 10 of the initial analysis was used. In addition, all images were batch-reanalyzed with one parameter value set for each tested chemical dose, referring to the parameter values of the individual reanalysis. The number of cells and MN cells counted by automatic image analysis using the MN/cell detection software are shown in Table 1. The cell number for each dose sample in the individual reanalysis was comparable to the total cell number obtained with manual counting or initial analysis, with a difference of less than 50. In addition, as the number of MN cells in each sample was corrected, the significant difference in the number of MN cells, which was observed in the initial analysis between manual counting, was no longer observed between the individual reanalysis and manual counting (Table 1, Table S2).

Microphotographs were reanalyzed using various thresholds. The optimal threshold value for individual reanalysis differed slightly for each individual image; however, MN detection was optimized in many images by adjusting Micronucleus TH. If the MN cell count was still not optimized, it was improved by increasing the Binarization TH or by slightly increasing the noise reduction applied at a value of 21 or 11. The difference in the MN frequency (%) detected by manual measurement and image analysis was approximately 1% for both samples (Table 1). The coefficient of determination between manual counting and individual reanalysis in detecting MN cell counts was 0.845, an improvement compared to the coefficient of determination between manual counting and the initial analysis (*R*²=0.624) (Fig. 4E). The results of batch reanalysis were numerically closer to those obtained by manual counting. However, the coefficient of determination between manual and batch reanalysis was only 0.666 (Fig. 4F).

## Discussion

In this study, CHL/IU cells were incubated with the test substance for 24 h, and a micronucleus test was performed without adding the S9 fraction of the liver homogenate supernatant. The number of micronuclei in the control cells was 7/1000 as calculated by manual measurement and 8/1000 as calculated by image analysis. This was approximately equal to the values reported in the literature [18], in which the number of micronuclei generated in CHIL/IU cells cultured for 24 h in the absence of S9 was 5/1000 cells. The cells that had an additional 24-h recovery period after drug treatment remained attached to the glass slides after PBS rinsing for cell fixation, and no partially broken cells were found in the field of view within the range of reagent concentrations referred to in published data [18, 19]. Results obtained by manual counting showed that the number of micronuclei generated in cells treated with MMC, MMS, and H_2_O_2_ followed a trend similar to previously published results [18]. Specifically, MMC and MMS exhibited peak micronucleation at doses of 0.1 µg/mL and 40 µg/mL, respectively. At the highest tested dose, the reagents exhibited cytotoxicity. When 80 µg/mL of MMS was administered to cells, cell proliferation was suppressed, and the number of cells visible in the field of view was significantly reduced, necessitating an increase in the number of photo images taken. The relative viability of MMS-treated cells decreased in a dose-dependent manner; however, it was different in the case of MMC-treated cells. Specifically, the relative survival rate exceeded 100% in the 0–0.05 µg/mL range, and the survival rate was higher at 0.2 µg/mL than at 0.1 µg/mL (Table 1, Fig S1). It has been reported that the relative viability of CHL/IU cells at MMC concentrations of 0–10,000 ng/mL or 0–22,200 ng/mL was higher than that at the zero or low doses, decreased owing to cytotoxicity at higher doses, with two to three peaks of viability observed within this dose range [20]. It was suggested that MMC caused a hormetic effect that increased relative survival at low doses and multiple survival peaks due to cellular responses, such as repair and apoptosis [20], which may have appeared in our micronucleus test.

In contrast, H_2_O_2_ increased the number of micronuclei generated in a dose-dependent manner, as previously described [18]. In our experiment, the cells were administered at 3.75, 7.5, 15, and 30 µg/mL, which were threefold higher than those reported in the literature; however, even at the highest concentration of 30 µg/mL, the cells survived and increased MN frequency to about 10% (Table 1). When K_2_CrO_4_ was administered to the CHL/IU cells, micronuclei were generated in a concentration-dependent manner, even at low concentrations. The cells treated with the highest dose of 4.9 µg/mL showed the highest number of micronuclei. The report on the exposure of human lymphocytes to K2CrO4 showed that a low concentration of 25 µM (4.83 µg/mL) for 2 h was not genotoxic to the interphase and metaphase chromatin compared to cadmium. However, a higher concentration of 150 µM was genotoxic than cadmium [19]. There were a few differences between a previous report and our study regarding the cell type, the administration period of K_2_CrO_4_, and the concentration, which was equivalent to the high concentration in our experiments, was low. However, it could be said that the previous report supported our results of CHL/IU cells treated with the 0–4.9 µg/mL concentration range of K_2_CrO_4_ for 24 h, which increased the number of micronuclei.

The cell staining conditions are significant factors in the performance of our software, and evenly stained specimens yielded accurate micronucleus incidence results. In this study, to measure all cells in the imaging field under a microscope, the cells cultured on the glass slide were fixed with 2% PFA at neutral pH after incubation with the test substance without trypsin treatment (Fig. 3). Even when adherent cells were trypsinized according to conventional methods [21], the cells were slightly swollen and fixed with 2% PFA, permeabilized with Triton, and stained with AO dye (Fig S3). If both the AO-stained cytoplasm and cell nucleus are green, this may be because the AO concentration is too low [22] or the fluorescence lifetime of the dye is short. In such cases, it is better to re-stain with a new dye. However, if the cells are stained too intensely such that not only the cytoplasm but even the cell nucleus turns red, washing the cells with PBS will remove the excess dye, and the cytoplasm and cell nucleus coloring will be correct.

The results of the first automated image analysis showed that the total number of cells detected was approximately equal to the manual counts (Table 1; Fig. 4A), demonstrating that there were no problems with cell counting using this software. In contrast, the detection rate of MN cells in the initial analysis was approximately 60% of that obtained with manual counting (Fig. 4D). It was thought that the uneven staining of the cytoplasm was accentuated during the image analysis process, resulting in high-intensity spots that were easily detected as pseudo-small nuclei.

When using the individual reanalysis mode of the software, the micronucleus TH of 0.6 to 0.7 improved the detection of MN cells in many target images, and with subsequent adjustment of the binarization TH, the detection accuracy was better (Fig. 4E). Although these threshold changes might have increased or decreased the total cell number by a few cells, the effect was small (Fig. 4B) and mainly helped improve the MN cell number.

Although there was no significant difference in the results of the batch reanalysis compared to the manual counting results for either the total cell count or the MN cell count (Table 1), the accuracy of the detection of MN cell counts was approximately 60%, which was similar to that of the first analysis (Fig. 4F). In batch reanalysis, a single binarization TH was reapplied to a group of images with multiple binarization THs (65, 75, 85, 120, and 165) assigned during the initial analysis. Consequently, it was thought that the inappropriate binarization of the TH led to erroneous position detection of the MN in some images. On the other hand, the images of cells treated with 1.2 µg/mL of K_2_CrO_4_ and 40 µg/mL of MMS had been given identical binarization TH in each image series at the initial analysis (Table S1), the numbers of total cells and the MN cells in the batch reanalysis was similar to that of the manual counting or the initial analysis (Table 1), indicating that the parameters could be fine-tuned and still be adequately reanalyzed. In some cases, batch reanalysis improved the MN cell counts in groups of images, even with multiple binarization THs. However, collecting images in a folder with the same binarization TH for batch reanalysis is probably safer.

If the software mistakenly detects small foreign or cell debris as MN cells, the detection error may be improved by increasing the value of micronucleus TH, which changes the sensitivity of detection based on the circularity of the MN. However, in the enlargement/reduction process for detecting cell nuclei, MNs that were in close contact with the cell nucleus were difficult to detect because they were quickly recognized as part of the shape of the cell nucleus. MN cells that could be visually determined but were not detected by this software were considered accurate for detection. If the detection position of the MN cells is inappropriate, the binarization TH, which determines the cytoplasmic region, must be corrected. In the initial analysis, one of the five specified values (65, 75, 85, 120, and 165) was assigned to each image as the binarization TH, depending on the histogram value of the green image; however, other intermediate values may be suitable. Because batch reanalysis updates these binarized THs with a single value, it is better to reanalyze only images with the same binarized TH.

The following is a brief description of the method employed by this software. Before a large number of micronucleus test images are analyzed, the software automatically analyzes a few representative images in the initial analysis and then determines the optimal threshold value in the individual reanalysis mode. Subsequently, an initial analysis is performed with a large number of images. It is recommended that the images should be collected with the same binarization TH after the initial analysis or be pre-separated into images with similar cell staining intensity, color, and brightness before the initial analysis. Finally, batch reanalysis of each image is performed using the six parameter values found in the individual reanalysis mode. In addition, it may be possible to achieve counting reproducibility by preparing a sample image in which the cytoplasm and MN positions and numbers can be detected correctly, which will enable parameter values to be easily obtained in advance.

The software processing speed depends on the computer processing power; therefore, a computer equipped with a high-speed CPU, RAM with high capacity, and the latest OS can perform image analysis at a high speed. Our software was developed as a counting tool that can run on Windows 10; however, we confirmed that it can also run on Windows 11. It took approximately 10 min to analyze approximately 50 images on a laptop PC with Windows 10 (approximately 30 s per image in the individual reanalysis), and the analysis time was shorter by approximately 5 min on a desktop PC with 32 MB of RAM and Windows 11 (the reanalysis time in individual mode was even shorter).

This report does not provide results that compare the performance of this software with other analysis applications; however, we tested it using a few sample images. CellProfiler [10] was able to split color images into RGB and detect the number of cytoplasm and MNs with pipeline settings; however, the analysis speed was several minutes per image (Fig S4). In ImageJ [17], unless the analysis processes of cell number calculation and micronucleus number calculation are conducted separately, the processing is complicated, and macros are prone to errors. It is also complex and challenging to create pipelines and macroscopes that count the number of MN cells with overlapping micronuclei and cytoplasmic locations, apart from the total number of cells. In this respect, our MN/cell detection software can detect a single MN cell even if there are two or more MNs in the cytoplasm, and the acceptable image-processing speed is an advantage that other free applications do not have. Our software performs an initial analysis to obtain the parameter values for each image. However, if the results are unsatisfactory, it provides two reanalysis modes: individual reanalysis, which adjusts parameters for each image, and batch reanalysis, which analyzes many images using arbitrary threshold settings.

Many micronucleus detection applications developed in recent years include advanced tools that use deep learning to automatically classify cell nuclei based on their shape [23–25]. As many studies have used lymphocytes as test cells, these methods may also be applied to adherent cells that have been fixed and have a round shape after trypsin treatment. The next challenge is that this software currently needs to be compatible with CBMN assays, which require the shape classification of cell nuclei. However, it is suitable for routine micronucleus testing, student-level experiments, and the simultaneous testing of unknown test substances at no cost. Because this software runs as a simple program, it is easy to maintain and improve the software system. We hope that this software will reduce the burden of conducting micronucleus tests on experimenters.

## Conclusions

Our newly developed MN/cell detection software enables high-speed image processing and analysis without requiring high computer processing power. Furthermore, the data obtained was almost comparable to those obtained by manual MN counting.

### Supplementary Information

**Additional file 1: Fig S1.** Cytotoxicity of tested chemicals. **Fig S2.** Representative micrographs of cells with different binarization thresholds each other. **Fig S3.** The auto-image analysis of captured images of cells treated with trypsin, the fixation using acetate/methanol, and the staining with acridine orange. **Fig S4.** Example of micronucleus detection using CellProfier. **Supplemental Table 1.** The parameter values of the initial analysis of sample images obtained by the MN/cell detection software. **Supplemental Table 2.** Result of Student’s *t*-test (one sided test).

## Data Availability

The datasets generated and analyzed during the current study are available from the corresponding author upon reasonable request.
