# Supplementary material for: Application of image-recognition techniques to automated micronucleus detection in the in vitro micronucleus assay
Source: Genes Environ. 2024 Apr 24;46:11. doi: 10.1186/s41021-024-00305-9 (PMC11040892; doi:10.1186/s41021-024-00305-9)
Supplement: Supplementary file 1 — Additional file 1: Fig S1. Cytotoxicity of tested chemicals. Fig S2. Representative micrographs of cells with different binarization thresholds each other. Fig S3. The auto-image analysis of captured images of cells treated with trypsin, the fixation using acetate/methanol, and the staining with acridine orange. Fig S4. Example of micronucleus detection using CellProfier. Supplemental Table 1. The parameter values of the initial analysis of sample images obtained by the MN/cell detection software. Supplemental Table 2. Result of Student’s t-test (one sided test). [file 41021_2024_305_MOESM1_ESM.pdf]

*Supplementary information*

# Application of image-recognition techniques to automated micronucleus detection in the *in vitro* micronucleus assay

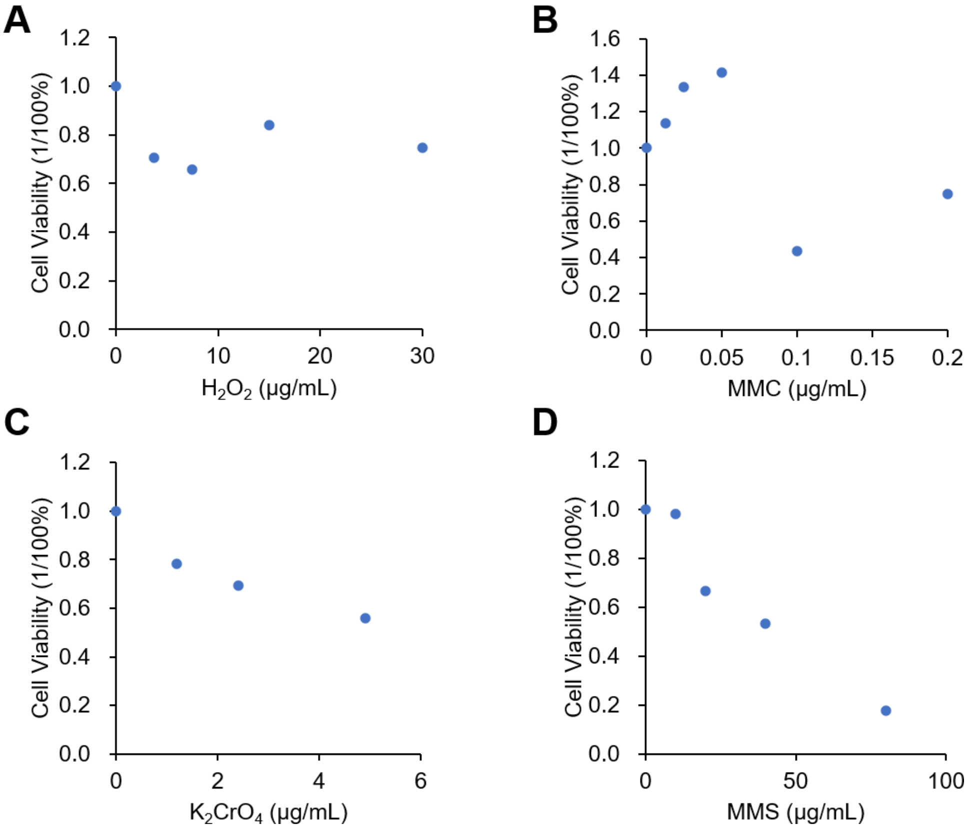


Fig S1, Cytotoxicity of tested chemicals.

A) $H_2O_2$, B) MMC, C) $K_2CrO_4$, D) MMS.

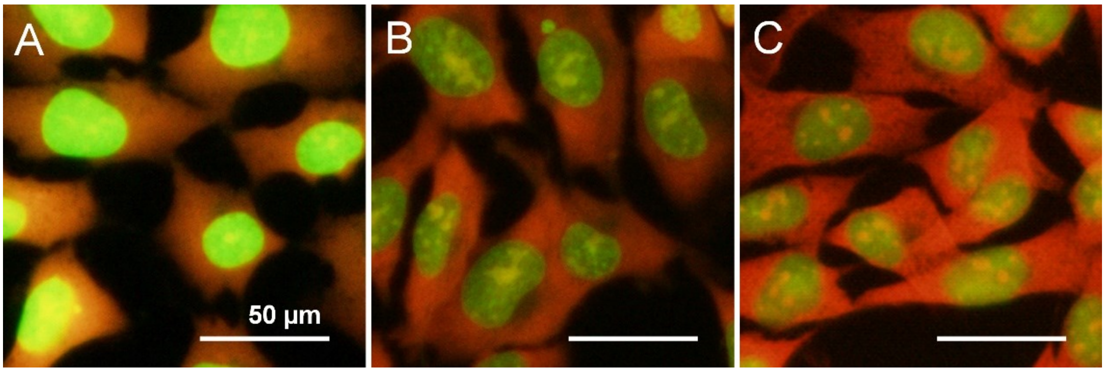


Fig S2. Representative micrographs of cells with different binarization thresholds each other. The binarization thresholds of the photos were (A) 165, (B) 92, and (C) 70. All micrograph bars showed 50 μm. The binarization thresholds of (B) and (C) were adjusted for illustrative purposes. (A) When the cytoplasm of the cells in the image was orange, and the cell nucleus was green with good coloration, the binarization threshold was 165. (B) When the cytoplasm was orange, the nucleus was green but dim, and the internal granules were visible, the value was 92. (C) When the cytoplasm was red and the nucleus was reddish green with visible internal granules, the value was 70. The more distinct the color difference between the cytoplasm and cell nucleus, the binarization threshold was the higher.

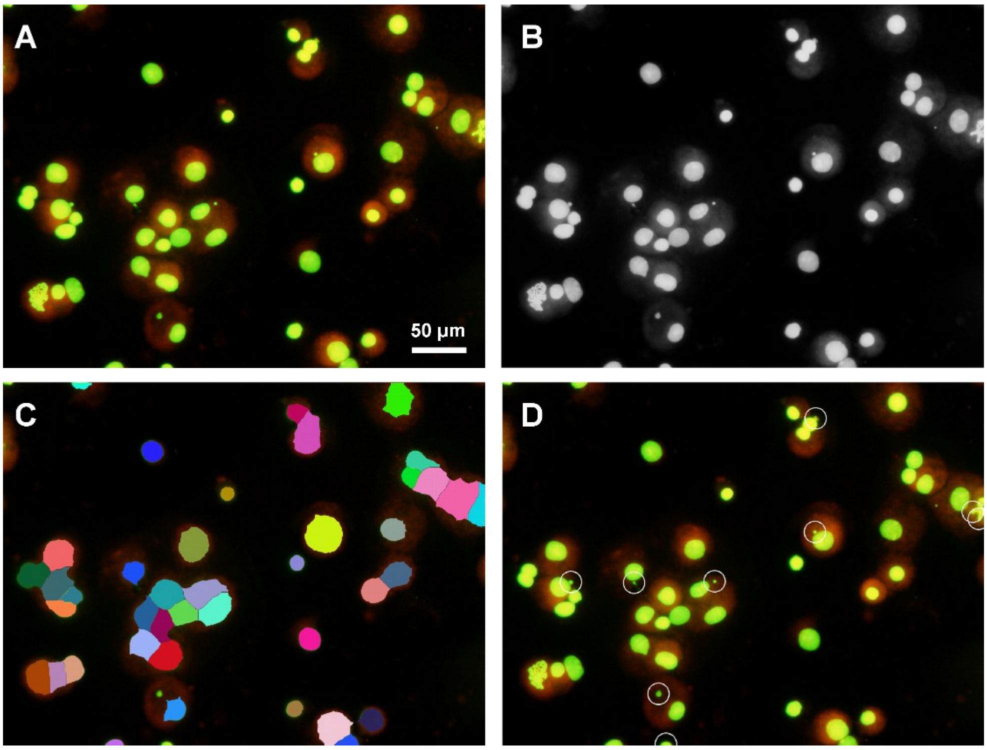


Fig S3. The auto-image analysis of captured images of cells treated with trypsin, the fixation using acetate/methanol, and the staining with acridine orange.

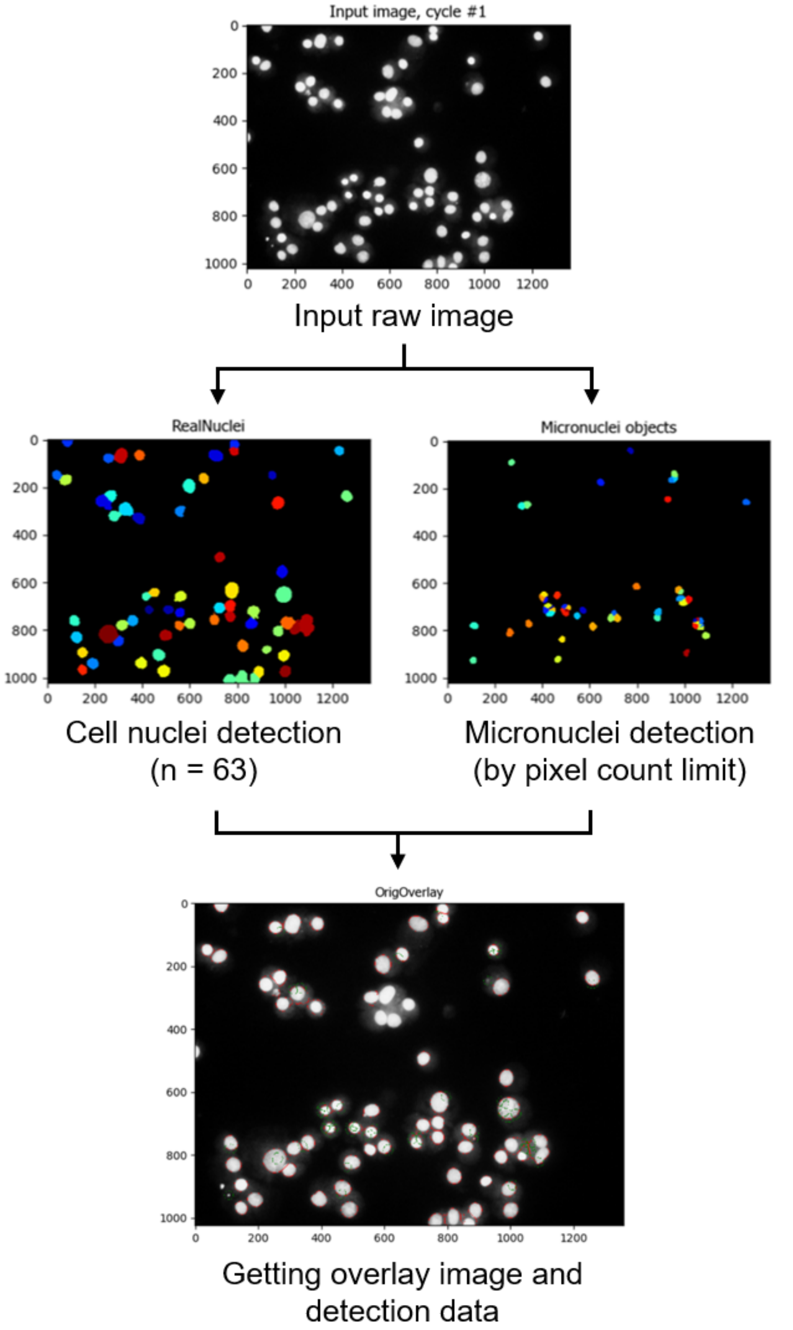


Fig S4, Example of micronucleus detection using CellProfier.

Supplemental Table 1. The parameter values of the initial analysis of sample images obtained by the MN/cell detection software.

| Sample | Conc. (μg/mL) | Number of micrograph | Binarization threshold | Kernel size | | Noise removal | Threshold for MN roundness | Tone center value |
|---|---|---|---|---|---|---|---|---|
| | | | | Small | Large | | | |
| Control | - | 45 | 120 (3), 165 (42) | 13 | 25 | 21 | 0.5 | 118 |
| $H_2O_2$ | 1.25 | 55 | 75 (13), 85 (27), 120 (15) | 13 | 25 | 21 | 0.5 | 118 |
| | 2.5 | 57 | 165 (57) | 13 | 25 | 21 | 0.5 | 118 |
| | 5 | 43 | 165 (43) | 13 | 25 | 21 | 0.5 | 118 |
| | 10 | 79 | 65 (37), 75 (19), 85 (20), 120 (3) | 7 | 13 | 11 | 0.5 | 118 |
| $K_2CrO_4$ | 1.2 | 50 | 165 (50) | 13 | 25 | 21 | 0.5 | 118 |
| | 2.4 | 60 | 165 (60) | 13 | 25 | 21 | 0.5 | 118 |
| | 4.9 | 66 | 120 (6), 165 (60) | 13 | 25 | 21 | 0.5 | 118 |
| MMC | 0.0125 | 49 | 165 (49) | 13 | 25 | 21 | 0.5 | 118 |
| | 0.025 | 36 | 65 (6), 75 (8), 85 (21), 120 (1) | 7 | 13 | 11 | 0.5 | 118 |
| | 0.05 | 30 | 85 (22), 120 (8) | 7 | 13 | 11 | 0.5 | 118 |
| | 0.1 | 80 | 65 (7), 75 (14), 85 (37), 120 (22) | 13 | 25 | 21 | 0.5 | 118 |
| | 0.2 | 65 | 65 (63), 75 (1), 85 (1) | 7 | 13 | 11 | 0.5 | 118 |
| MMS | 10 | 49 | 165 (49) | 13 | 25 | 21 | 0.5 | 118 |
| | 20 | 50 | 75 (1), 120 (5), 165 (44) | 13 | 25 | 21 | 0.5 | 118 |
| | 40 | 73 | 65 (73) | 7 | 13 | 11 | 0.5 | 118 |
| | 80 | 187 | 65 (71), 75 (37), 85 (60), 120 (19) | 13 | 25 | 21 | 0.5 | 118 |

The parentheses in the binarization threshold showed the number of applicable images.

Supplemental Table 2. Result of Student's *t*-test (one sided test)

| | | | Initial analysis vs manual counting | | | | Individual reanalysis vs manual counting | | | | Batch reanalysis vs manual counting | | | |
|---|---|---|---|---|---|---|---|---|---|---|---|---|---|---|
| | | | Total cells | | NM cells | | Total cells | | NM cells | | Total cells | | NM cells | |
| Sample | (µg/mL) | *df* | *t* | *p* | *t* | *p* | *t* | *p* | *t* | *p* | *t* | *p* | *t* | *p* |
| Control | 0 | 88 | -1.66 | 0.473 | 1.66 | 0.354 | 1.66 | 0.477 | 1.66 | 0.202 | -1.66 | 0.358 | 1.66 | 0.076 |
| $H_2O_2$ | 3.75 | 108 | 1.66 | 0.446 | 1.66 | <0.001 | 1.66 | 0.446 | -1.66 | 0.392 | 1.66 | 0.446 | -1.66 | 0.162 |
| | 7.5 | 112 | 1.66 | 0.298 | 1.66 | 0.042 | 1.66 | 0.475 | 1.66 | 0.373 | 1.66 | 0.308 | -1.66 | 0.246 |
| | 15 | 84 | 1.66 | 0.440 | -1.66 | 0.474 | -1.66 | 0.440 | -1.66 | 0.474 | 1.66 | 0.440 | -1.66 | 0.474 |
| | 30 | 156 | 1.65 | 0.467 | 1.65 | 0.021 | 1.65 | 0.462 | 1.65 | 0.469 | 1.65 | 0.367 | 1.65 | 0.469 |
| $K_2CrO_4$ | 1.2 | 98 | 1.66 | 0.495 | -1.66 | 0.249 | -1.66 | 0.495 | -1.66 | 0.286 | -1.66 | 0.473 | -1.66 | 0.327 |
| | 2.4 | 118 | -1.66 | 0.378 | -1.66 | 0.479 | -1.66 | 0.378 | 1.66 | 0.478 | -1.66 | 0.399 | -1.66 | 0.413 |
| | 4.9 | 130 | 1.66 | 0.458 | -1.66 | 0.037 | -1.66 | 0.483 | -1.66 | 0.350 | 1.66 | 0.381 | -1.66 | 0.189 |
| MMC | 0.0125 | 96 | -1.66 | 0.424 | 1.66 | 0.152 | 1.66 | 0.402 | 1.66 | 0.308 | -1.66 | 0.462 | 1.66 | 0.119 |
| | 0.025 | 70 | 1.67 | 0.443 | 1.67 | 0.005 | 1.67 | 0.452 | 1.67 | 0.421 | 1.67 | 0.415 | -1.67 | 0.312 |
| | 0.05 | 58 | -1.67 | 0.463 | 1.67 | 0.144 | 1.67 | 0.463 | -1.67 | 0.483 | 1.67 | 0.375 | -1.67 | 0.232 |
| | 0.1 | 158 | -1.65 | 0.328 | -1.65 | 0.426 | -1.65 | 0.329 | -1.65 | 0.344 | -1.65 | 0.384 | -1.65 | 0.289 |
| | 0.2 | 128 | -1.66 | 0.492 | 1.66 | 0.064 | 1.66 | 0.483 | -1.66 | 0.454 | 1.66 | 0.419 | -1.66 | 0.477 |
| MMS | 10 | 96 | -1.66 | 0.442 | -1.66 | 0.321 | -1.66 | 0.431 | -1.66 | 0.082 | -1.66 | 0.460 | 1.66 | 0.453 |
| | 20 | 98 | -1.66 | 0.490 | 1.66 | 0.392 | 1.66 | 0.481 | 1.66 | 0.369 | -1.66 | 0.387 | 1.66 | 0.436 |
| | 40 | 144 | 1.66 | 0.417 | 1.66 | 0.407 | 1.66 | 0.438 | -1.66 | 0.354 | 1.66 | 0.427 | 1.66 | 0.407 |
| | 80 | 372 | 1.65 | 0.370 | 1.65 | <0.001 | 1.65 | 0.370 | 1.65 | 0.427 | 1.65 | 0.459 | 1.65 | 0.220 |
